# Supplementary material for: IFNα gene/cell therapy curbs colorectal cancer colonization of the liver by acting on the hepatic microenvironment
Source: EMBO Mol Med. 2016 Jan 14;8(2):155–70. doi: 10.15252/emmm.201505395 (PMC4734840; doi:10.15252/emmm.201505395)
Supplement: Supplementary file 1 — Appendix [file EMMM-8-155-s001.pdf]

Appendix

IFNα gene/cell therapy curbs colorectal cancer colonization of the liver by acting on the hepatic microenvironment

Mario Catarinella, Andrea Monestiroli, Giulia Escobar, Amleto Fiocchi, Ngoc Lan Tran, Roberto Aiolfi, Paolo Marra, Antonio Esposito, Federica Cipriani, Luca Aldrighetti, Matteo Iannacone, Luigi Naldini, Luca G. Guidotti and Giovanni Sitia

Table of contents

	Page
**Appendix Figure S1**	**2-3**
**Appendix Figure S2**	**4-5**
**Appendix Figure S3**	**6**
**Appendix Figure S4**	**7-8**
**Appendix Figure S5**	**9-10**
**Appendix Figure S6**	**11-12**
**Appendix Supplementary Materials and Methods**	**13-24**
**Appendix References**	**25-26**

Appendix Figure S1

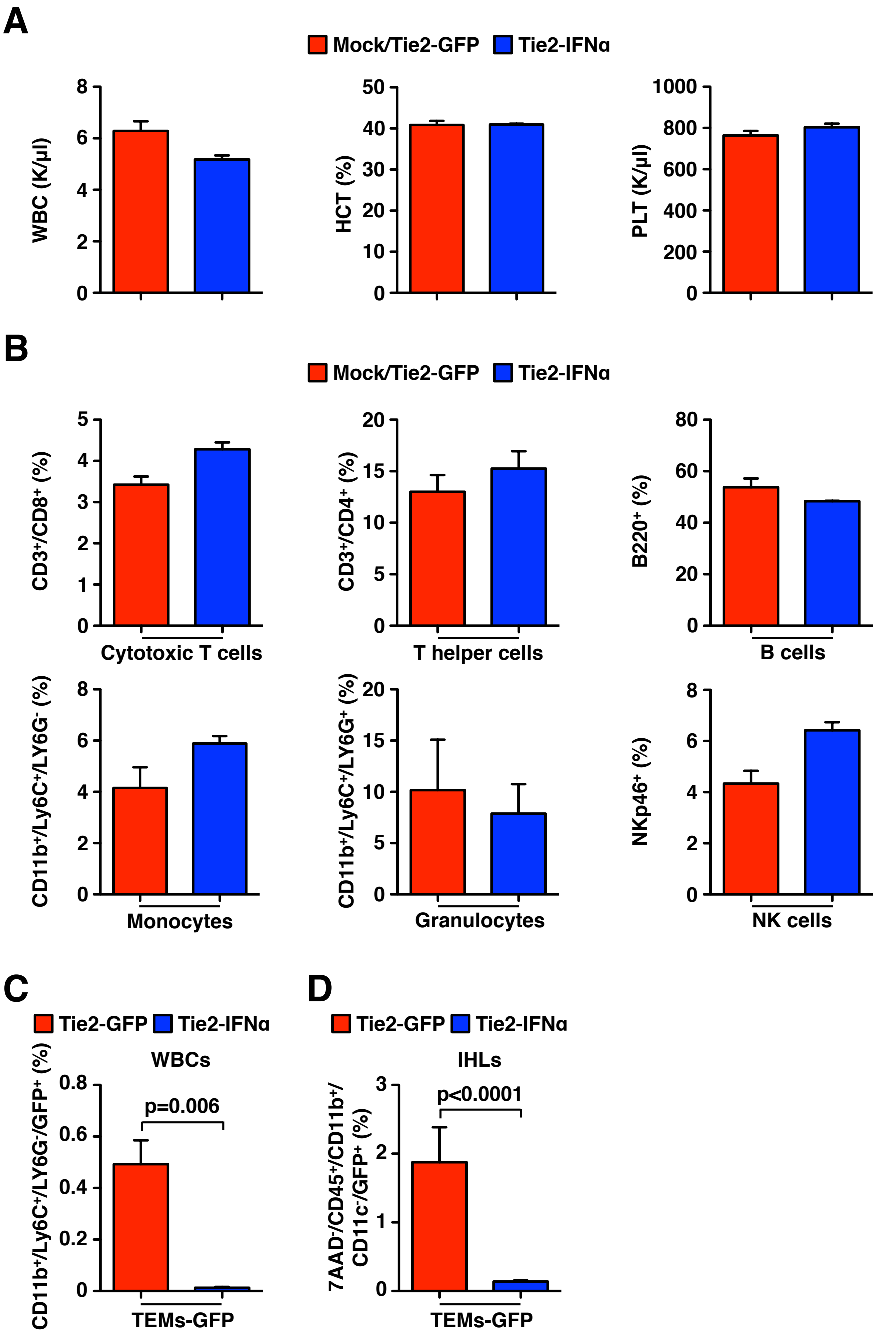

Appendix Figure S1. Analysis of bone marrow reconstitution in transplanted mice. HSPCs purified from BM of syngeneic donor mice were enriched and transduced as previously described (see Appendix Supplementary Materials and Methods for details). Seven to 10 weeks post-transplant, recipient mice were bled and analyzed for BM reconstitution.

A. White blood cell (WBC), hematocrit (HCT) and platelet (PLT) counts from

reconstituted mice (Mock/Tie2-GFP n=15/n=41 respectively, VCN=3.21±0.16; Tie2-IFNα n=38, VCN=1.00±0.01); mean values and S.E.M. of three independent transplants are shown; differences were not statistically significant by unpaired Student's t-Test.

B. Flow cytometry analysis of WBCs from mice transplanted and reconstituted as above. Cell population and corresponding gating strategy are indicated. %=percentage of total WBCs; mean values and S.E.M. of two (NK cells, cytotoxic T cells, T helper cells and B cells; Tie2-GFP n=17, VCN=1.46±0.01; Tie2-IFNα n=20, VCN=0.55±0.01) or three (monocytes and granulocytes; Mock/Tie2-GFP n=53, VCN=3.21±0.16; Tie2-IFNα n=37, VCN=1.00±0.01) independent transplants are shown; differences were not statistically significant by unpaired Student's t-Test.

C. Flow cytometry characterization of TEMs-GFP within total WBCs of Tie2-GFP mice (n=41) described in **A**. WBCs isolated from the blood of Tie2-IFNα mice were utilized to estimate the GFP background signal. Mean values and S.E.M. are shown; indicated *p*-value was calculated by unpaired Student's t-Test.

D. Flow cytometry characterization of TEMs-GFP within the IHLs of Tie2-GFP mice (VCN=2.74±0.52) transplanted and reconstituted as above and intrasplenically injected with NaCl (n=3). Tie2-IFNα mice were utilized to estimate the GFP background signal. Mean values and S.E.M. are shown; indicated *p*-value was calculated by unpaired Student's t-Test.

Appendix Figure S2

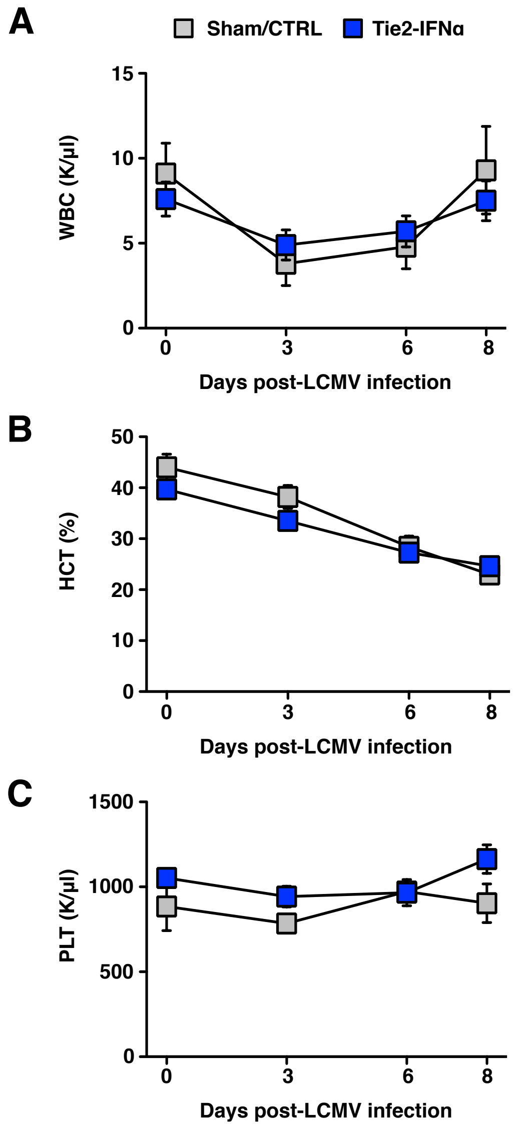

Appendix Figure S2. Basic hematological values of Tie2-IFNα mice and Sham/CTRL mice infected with LCMV.

A to C. White blood cell (WBC), hematocrit (HCT) and platelet (PLT) counts from LCMV infected mice (Armstrong strain, 200 pfu intraperitoneally injected) described in Fig 2F. Sham=mice transplanted with non-transduced HSPCs and intrasplenically injected with NaCl; CTRL=wild-type CB6 control mice. Note that at this low-dose infecting condition, LCMV viremia - which peaks by day 4 and disappears by day 7-8 post-infection when larger inocula are utilized experimentally (Iannacone et al, 2008) - remained undetectable throughout the course of the infection. No significant differences between the two groups were observed (by unpaired Student's t-Test). The decrease of HCT values observed in both Sham/CTRL and Tie2-IFNα mice represents a transient effect caused by the high frequency of blood withdrawal. Shortly after the end of the experiment the HCT values of both groups returned and remained to normal levels

throughout the animals lifespan (as depicted in Fig EV2A). Data pooled from two independent experiments, Sham/CTRL (n=6), Tie2-IFNα (n=7); mean values are shown; error bars=S.E.M.

Appendix Figure S3

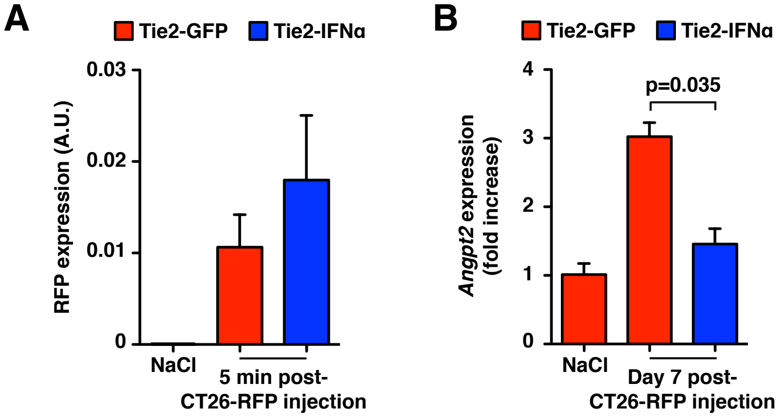

Appendix Figure S3. Molecular analysis of CRC cell arrival and tumor-driven angiogenesis analysis in the liver of Tie2-GFP and Tie2-IFNα mice.

A. Quantitative real-time PCR analysis of CT26-RFP arrival in the liver of Tie2-GFP (n=9) or Tie2-IFNα (n=5) transplanted mice, 5 minutes post-intrasplenic injection of $5x10^5$ CT26-RFP. Data pooled from three independent experiments; mean values are shown; error bars=S.E.M.; differences were not statistically significant by Mann-Whitney test.

B. Relative mRNA expression analysis of the pro-angiogenic gene *Angpt2* in the liver of Tie2-GFP (n=5) or Tie2-IFNα (n=7) mice, 7 days post-intrasplenic injection of $5x10^5$ CT26-RFP. The basal expression of *Angpt2* was estimated by quantitative real-time PCR in control mice (i.e. Tie2-GFP injected with saline; n=2), set to 1 as reference value and utilized to calculate the fold increase values at day 7 post-injection. Data pooled from two independent experiments; mean values are shown; error bars=S.E.M.; indicated *p*-value was calculated by Mann-Whitney test.

Appendix Figure S4

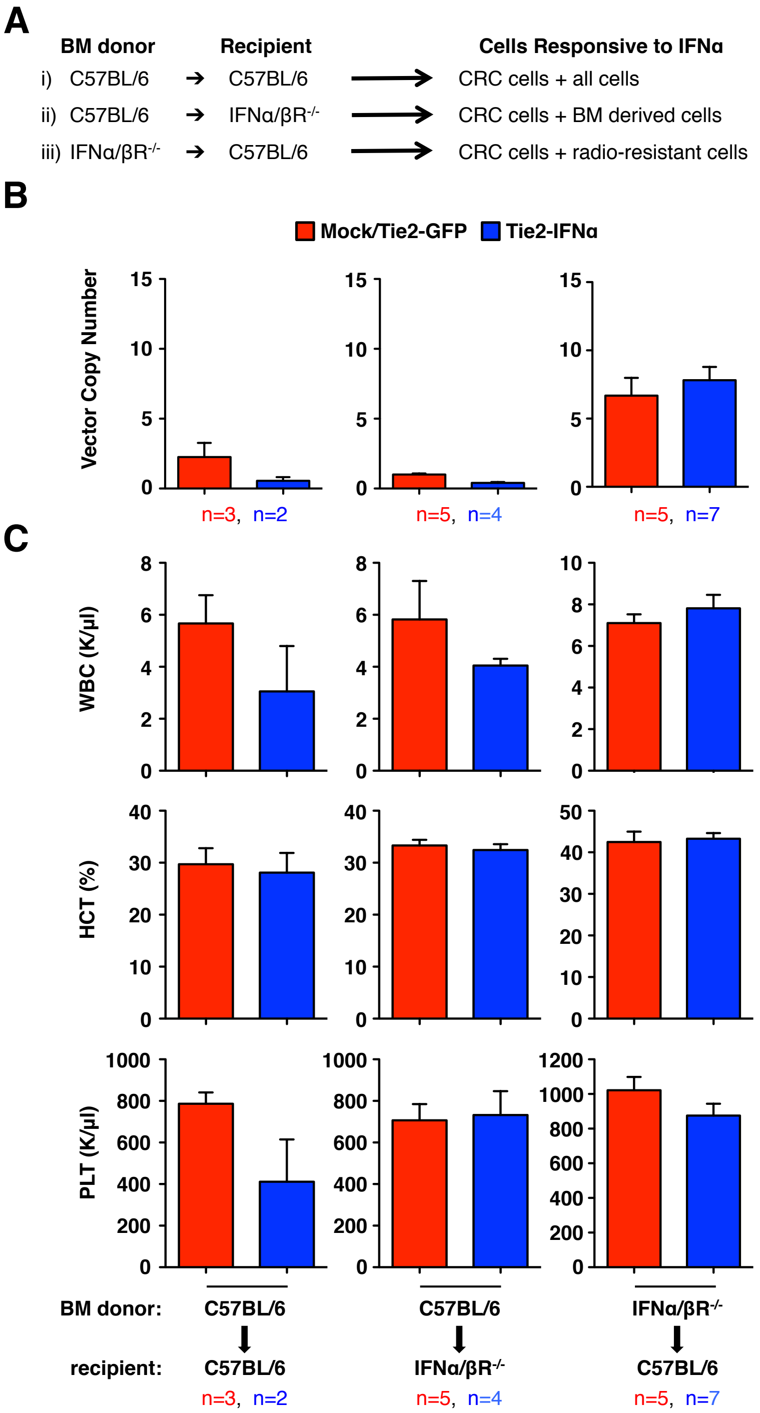

Appendix Figure S4. Basic hematological values of IFNα/β receptor knock out (IFNα/βR$^{-/-}$) BM chimeric mice.

A. To identify the primary targets of Tie2-IFNα, reciprocal BM transplantations between IFNα/βR$^{-/-}$ mice and syngeneic C57BL/6 mice were made (BM donor from which HSPCs were purified and recipient mouse strains are listed on the left). The resulting chimeric

animals had restricted cellular compartments responsive to IFNα that could be analyzed (indicated on the right after the long arrows). HSPCs purification, transduction and transplantation were performed as previously described for CB6 mice.

B. Quantification of vector copy number (VCN) performed by quantitative real-time PCR on white blood cells extracted from the chimeric mice described in **A**, 7 weeks post-transplant. n=number of transplanted mice per group; BM donor and recipient mouse strains are indicated at the bottom of panel **C**; data pooled from three independent experiments; mean values are shown; error bars=S.E.M.

C. White blood cells (WBC, top panels), hematocrit (HCT, middle panels) and platelet counts (PLT, bottom panels) of the chimeric mice described in **B**. Note that as expected, both Tie2-GFP and Tie2-IFNα mice did not display significant differences (by unpaired Student's t-Test) in their basic hematological values even in C57BL/6 mice that received IFNα/βR$^{-/-}$ transduced HSPCs despite the higher VCN, confirming the insensitivity of IFNα/βR$^{-/-}$ HSPCs to a cytokine such as IFNα that has potential to induce BM aplasia (Binder et al, 1997). BM donor and recipient mouse strains and number of transplanted mice per group are shown; data pooled from three independent experiments; mean values are shown; error bars=S.E.M.

Appendix Figure S5

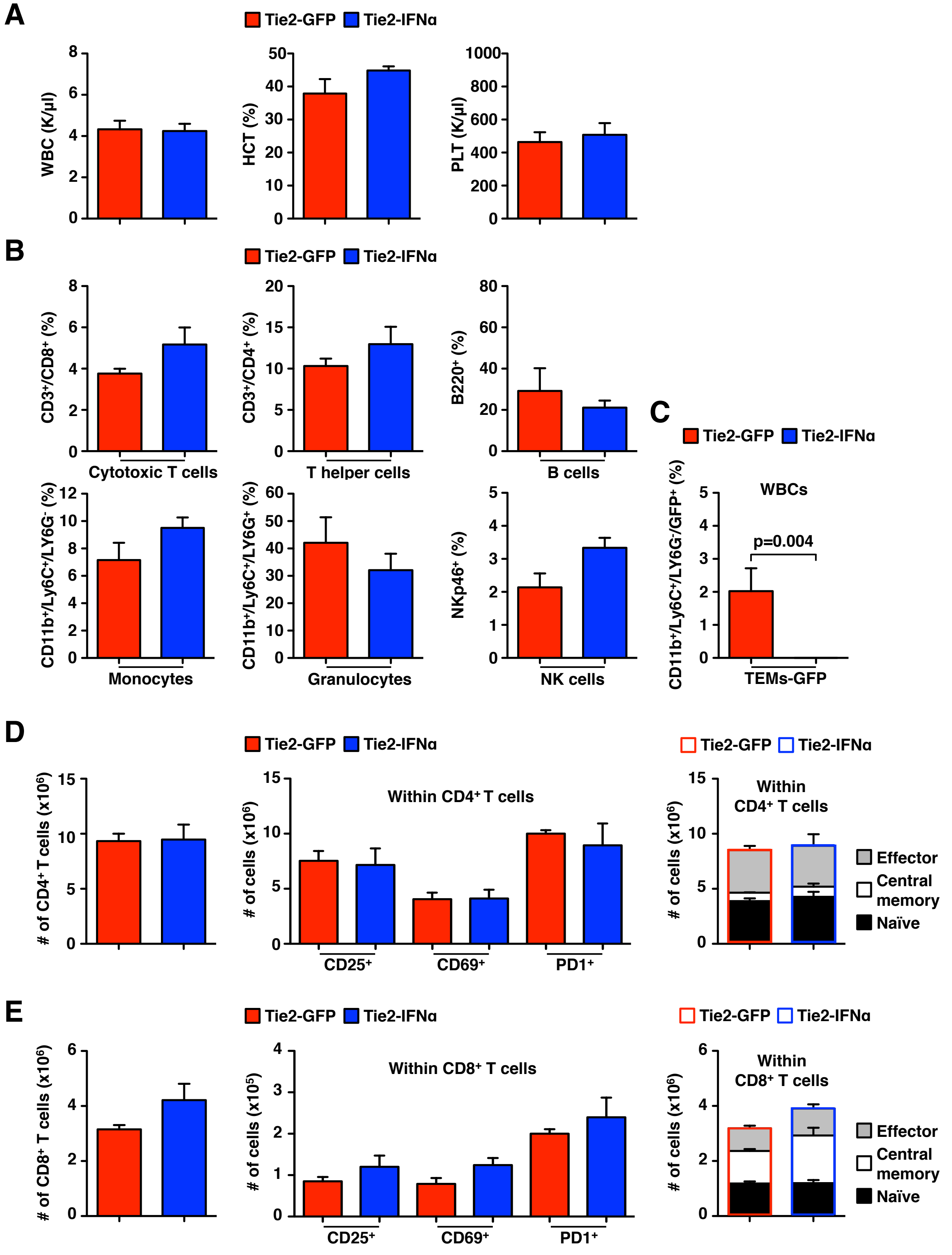

Appendix Figure S5. Analysis of bone marrow reconstitution and immune cell activation in transplanted mice with established CRC liver metastases.

HSPCs purified from BM of syngeneic donor mice were enriched and transduced as previously described (see Appendix Supplementary Materials and Methods for details). Eight days before transplantation, $5x10^3$ CT26 cells were injected under the liver capsule of anesthetized recipient mice. Twenty-eight days post-CT26 injection, recipient mice were bled and analyzed for BM reconstitution.

A. White blood cell (WBC), hematocrit (HCT) and platelet (PLT) counts from HSPCs reconstituted mice (Mock/Tie2-GFP n=7, VCN=16.82±3.15; Tie2-IFNα n=10, VCN=3.05±0.66); mean values and S.E.M. of two independent transplants are shown; differences were not statistically significant by unpaired Student’s t-Test.

B. Flow cytometry analysis of WBCs from the same mice described above. Cell population and corresponding gating strategy are indicated. %=percentage of total WBCs; mean values and S.E.M. of one (NK cells, cytotoxic T cells, T helper cells and B cells; Tie2-GFP n=4, VCN=12.85±0.96; Tie2-IFNα n=4, VCN=1.73±0.20) or two (monocytes and granulocytes; Mock/Tie2-GFP n=7, VCN=16.82±3.15; Tie2-IFNα n=10, VCN=3.05±0.66) independent transplants are shown; differences were not statistically significant by unpaired Student’s t-Test.

C. Flow cytometry characterization of TEMs-GFP within total WBCs of Tie2-GFP mice (n=7) described in **A**. WBCs isolated from the blood of Tie2-IFNα mice were utilized to estimate the GFP background signal. Mean values and S.E.M. are shown; indicated *p*-value was calculated by unpaired Student’s t-Test.

D, E. Flow cytometry characterization of immune $CD4^+$ T cells (**D**) and $CD8^+$ T cells (**E**) cells within splenocytes of Tie2-GFP mice (n=4) and Tie2-IFNα mice (n=4) described above. Left panels depict absolute numbers (#) of total $CD4^+$ and $CD8^+$ T cells. Early activation markers such as CD25, CD69 and PD1 are used to characterize the number of activated cells within either $CD4^+$ or $CD8^+$ T cells in both groups of animals (center panels). Right panels show absolute number of the indicated subpopulations of cells within either $CD4^+$ or $CD8^+$ splenocytes. Naïve $CD4^+$ or $CD8^+$ T cell populations are defined as either $CD4^+$ or $CD8^+$ T cells which are also $CD44^-$/$CD62L^+$; central memory $CD4^+$ or $CD8^+$ T cell populations are defined as either $CD4^+$ or $CD8^+$ T cells which are also $CD44^+$/$CD62L^+$; effector $CD4^+$ or $CD8^+$ T cell populations are defined as either $CD4^+$ or $CD8^+$ T cells which are also $CD44^+$/$CD62L^-$. Mean values and S.E.M. are shown; differences were not statistically significant by unpaired Student’s t-Test.

Appendix Figure S6

A

	Age (years)	Sex	CRC Stage (UICC-TNM 7th)	Grade	Chemotherapies	Recurrence (months)	Number of lesions	Dimension (cm)
Ctrl	51	M	NL	-	-	-	-	-
Pz-1	62	M	IV	G3	FOLFOX + bev	5	3	3,5
Pz-2	60	M	IV	G2	cape + bev	20	4	8,5
Pz-3	59	M	IV	G2	XELOX	9	1	7
Pz-4	73	M	I	G2	NO	65	1	1,5
Pz-5	63	M	IV	G2	FOLFOX + bev	-	4	8,5
Pz-6	82	M	IV	G2	XELOX	-	3	5,8
Pz-7	43	F	IV	G2-3	FOLFIRI + bev	13	6	3,4
Pz-8	60	F	IIIB	G2	FOLFIRI + bev	61	1	6

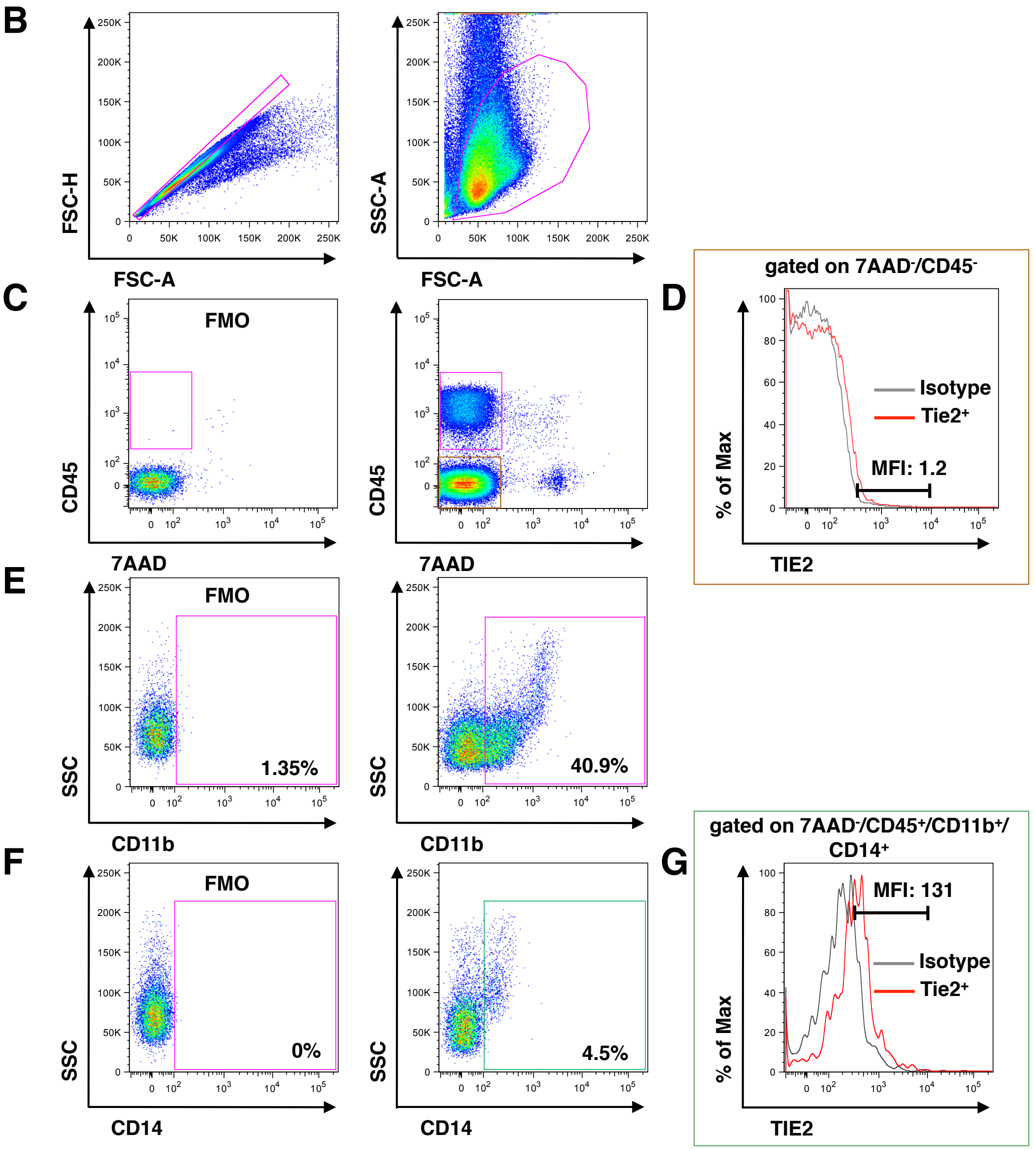

Appendix Figure S6. Clinical characteristics of the patients enrolled in the study, and liver TEMs gating strategy.

A. Clinical characteristics of the patients enrolled in the study at the time of liver resection. CRC staging was determined according to the guidelines of the Union for International Cancer Control (Sobin & Compton, 2010).

Recurrence: elapsed time between primary tumor and metastasis appearance; Dimension: sum of lesion maximal diameters; Ctrl: control, represented by a patient with hepatic hemangioma; NL: normal liver; Cape: capecitabine; Bev: bevacizumab; FOLFIRI: infusional 5-FU/bolus folinic acid plus irinotecan; Cet: cetuximab; XELOX: capecitabine plus oxaliplatin; FOLFOX: infusional 5-FU/bolus folinic acid plus oxaliplatin.

B to G. Representative gating strategy for the identification of TEMs in human liver. IHLs were extracted from liver resections. The gating strategy was performed as follows:

B. Physical parameters (pink gates).

C. $7AAD^-/CD45^-$ population (right panel, orange gate), $7AAD^-/CD45^+$ leukocytes (right panel, pink gate) and corresponding CD45 Fluorescence Minus One (FMO) staining (left panel, pink gate).

D. Histogram gated on the $7AAD^-/CD45^-$ population (orange gate) showing minimal Tie2 signal (red line), comparable to the corresponding isotype control (grey line).

E. $CD11b^+$ myeloid cells gated on $7AAD^-/CD45^+$ leukocytes (right panel, pink gate) and corresponding FMO staining (left panel, pink gate).

F. $CD14^+$ monocytes gated on $7AAD^-/CD45^+/CD11b^+$ leukocytes (right panel, green gate) and corresponding FMO staining (left panel, pink gate).

G. Histogram gated on the $7AAD^-/CD45^+/CD11b^+/CD14^+$ population (green gate) showing a higher intensity of Tie2 signal (red line) which identifies TEMs, compared to the corresponding isotype control (grey line).

The percentages depicted are calculated over the total $CD45^+$ population in panels **D** and **E**; the mean fluorescent intensity (MFI) values of $Tie2^+$ cells depicted in panels **C** and **F** were normalized by subtracting the background signal of the corresponding IgG isotype control.

Appendix Supplementary Materials and Methods

Colorectal cancer cell lines

Cells were cultured under standard condition at 37°C in a humid atmosphere with 5% CO_2 in RPMI medium (Gibco) supplemented with 10% FBS (Lonza) and 1% penicillin/streptomycin/L-glutamin (Gibco). The H-2d-restricted CT26 colorectal cancer cell line (Brattain et al, 1980) was purchased from American Type Culture Collection (ATCC). The H-2b-restricted MC38 colorectal cancer cell line (Rosenberg et al, 1986) was kindly provided by P. Berraondo López at the Centro de Investigación Médica Aplicada (CIMA) in Pamplona (Spain). Cell lines were routinely checked for mycoplasma contamination using the N-GARDE Mycoplasma PCR reagent set (EuroClone) and were not reauthenticated by the Authors. The *in vitro* sensitivity of CT26 or MC38 CRC cells towards recombinant murine IFNα1 (PBL Assay Science) was calculated with a standard *in vitro* MTT assay. Briefly, $4x10^3$ cell/well were plated in 96 wells flat bottom plate in either medium alone (RPMI-1640 [Gibco], 10% FBS [Lonza], 1% penicillin/streptomycin [Lonza]) or supplemented with the indicated doses of recombinant murine IFNα1 (from 500 to 2000 U/ml). Proliferation was measured by adding MTT (3-(4,5-dimethylthiazol-2-yl)-2,5-diphenyltetrazolium bromide, $1/10^{th}$ of culture volume) for 4 hours at 37°C. Once solubilized by dimethyl sulfoxide (DMSO) (Sigma-Aldrich) the absorbance was measured at 570 nm with a Multiskan Ascent 96 Plate Reader (MTX Lab System, Inc.). For selected experiments, CT26 were engineered by lentiviral transduction in order to stably express the red fluorescent protein (RFP) under the suCMV promoter (suCMV-RFP LVP309, AMSBIO).

Liver metastasis animal models

Eight to 10 weeks after HSPC transduction/transplantation, recipient mice were intrasplenically injected with different doses of CRC cells (either CT26, CT26-RFP or MC38) according to the experimental design. Intrasplenic CRC cell injection was carried out as follows: i) mice were anesthetized by intraperitoneal injection of tribromoethanol (250mg/kg, Avertin); ii) through a left lateral flank incision, spleens were surgically exteriorized and injected through the distal pole with the desired amount of CRC cells in either 100μl or 200μl of PBS depending on the cell dose; iii) 5 minutes post-injection spleens were surgically removed in order to avoid intrasplenic tumor growth; wounds were cauterized and sutured with two layers of silk 4/0 for the peritoneum and 7mm wound clips for the skin as previously described (Kuruppu et al, 1996). In experiments utilizing IFNα/βR$^{-/-}$ mice (inbred C57BL/6) or normal inbred C57BL/6 mice, only C57BL/6-derived MC38 cancer cells were used to avoid immune mediated rejection of mismatched cells.

In selected experiments, CRC cells were injected under the liver capsule as follow: i) mice were anesthetized by intraperitoneal injection of tribromoethanol (250mg/kg, Avertin); ii) through a midline incision, the left liver lobes were surgically exteriorized and 10μl of matrigel (BD Bioscience) containing 5x10^3 CT26 cells injected under the Glisson's capsule using a 29G needle as previously described (Kuo et al, 1995); iii) wounds were cauterized as described above (Kuruppu et al, 1996). Animals and biological samples were not anonymized for further analyses.

Plasmid constructs and LV production

Tie2-GFP and Tie2-IFNα_mirT plasmid constructs were generated as previously described (De Palma et al, 2003; Escobar et al, 2014). The Tie2-GFP_mirT construct was obtained by cloning the Wpre sequence and the mir-126 and -130a target sequences (SalI-KpnI) derived from Tie2-IFNα_mirT third generation LV in place of the Wpre sequence (SalI-KpnI) present in the third generation Tie2-GFP LV. Third-generation self inactivating LVs were produced in 293T cells by calcium phosphate transient transfection of the selected transfer vector, the packaging plasmid pMDLg/p.RRE, pCMV.REV, the VSV-G envelope plasmid pMD2.G and the pAdVAntage plasmid (Promega), as previously described (Escobar et al, 2014; Follenzi & Naldini, 2002). For vector titration 293T cells were transduced with serial vector dilutions in presence of polybrene (16µg/ml, Sigma-Aldrich). Genomic DNA (gDNA) was extracted 14 days after transduction and vector copies per diploid genome (vector copy number) were quantified by qPCR on 100ng of template gDNA using primers and a probe against the primer binding site region of LVs (HIV primer sense: 5' TACTGACGCTCTCGCACC-3'; HIV primer antisense 5'-TCTCGACGCAGGACTCG-3'; probe: FAM 5'-ATCTCTCTCCTTCTAGCCTC-3'). Endogenous DNA amount was quantified by a primers/probe set against the telomerase gene (Telo primer sense: 5'-GGCACACGTGGCTTTTCG-3'; Telo primer antisense: 5'-GGTGAACCTCGTAAGTTTATGCAA-3'; Telo probe: VIC 5'-TCAGGACGTCGAGTGGACACGGTG-3' TAMRA). Copies per genome were calculated by the formula=[ng LV/ng endogenous DNA]x[number of LV integrations in the standard curve]. The standard curve was generated using a CEM cell line stably carrying four

vector integrants previously determined by FISH and Southern Blot analyses. Each qPCR run carries an internal control generated using a CEM cell line stably carrying one vector integrant previously determined by FISH and Southern Blot analyses. Titer is expressed as Transducing Unit (TU)/ml and calculated using the formula TU/ml=(VCNx10^5x1/dilution factor).

Isolation and transduction of lineage negative hematopoietic stem/progenitor (HSPC)-enriched bone marrow cells (BM)

HSPC-enriched BM cells were isolated as previously described (Escobar et al, 2014) from CB6 mice, C57BL/6 mice or IFNα/βR$^{-/-}$ mice according to experimental needs. Briefly, total BM cells were isolated from femurs and tibias of 7 to 10 week-old donor mice via centrifugation and recovered in BM buffer (PBS [Euroclone], 2% FBS [Lonza], 1% penicillin/streptomycin [Lonza]). Cells were further isolated by density centrifugation gradient (82% Percoll and 18% Histopaque 1077; Sigma-Aldrich) and incubated with: 1) blocking solution (normal rat serum 4.5mg/ml of suspension, Abcam) 2) biotinylated anti-mouse Lineage panel (TER-119, clone Ter-119; Ly-6G/Ly-6C, clone RB6-8C5; CD3e, clone 145-2C11; CD45R, clone RA3-6B2; 30μg (each)/ml of suspension and CD11b, clone M1/70 0.25μg/ml of suspension; Biolegend) 3) bispecific tetrameric Ab complexes (100μl/ml of suspension; Stemcell Technologies); 4) colloidal magnetic dextran iron particles (60μl/ml of suspension; Stemcell Technologies). Each incubation was carried out for 15 minutes at 4°C. Lineage negative cells were separated utilizing the Magnetic StemSep Gravity Feed procedure (Stemcell Technologies) according to the manufacturer's instructions. The purified lineage negative cells were analyzed by

flow cytometry (biotin anti-mouse Lineage panel and Sca1, clone D7; Biolegend; c-Kit, clone 2B8; BD Biosciences) and utilized for lentiviral transduction only when HSPCs were enriched to more than 10% compared to the untreated total BM. Before transduction, $1.5x10^6$/ml HSPC-enriched cells were pre-incubated for 3 hours in StemSpan Serum-free medium (Stemcell Technologies), supplemented with 1% Pen/Strep/L-Glutamin (Lonza), 100ng/ml Stem Cell Factor, 100ng/ml FLT3 Ligand, 50ng/ml Thrombopoietin and 20ng/ml Interleukin-3 (Peprotech). Cells were then transduced for 12 hours with 10^8 TU/ml lentiviral particles (IFNα/$βR^{-/-}$ HSPC-enriched cells were transduced with 2.5 x 10^6 TU/ml lentiviral particles instead), recovered in PBS, counted and intravenously injected in the tail vein of lethally irradiated (700 Rad) recipient male mice ($1x10^6$ cells/mouse). Seven to 10 weeks post-transplant basic hematological values were measured and white blood cells isolated from peripheral blood of recipient mice were analyzed for: average number of lentiviral integrations per diploid genome (i.e. vector copy number [VCN]), GFP expression and percentage of WBC subsets (see peripheral blood cell analyses). As previously described (Escobar et al, 2014), VCN was also estimated on the outgrowth of a fraction of lineage negative HSPC-enriched cells (InVCN) cultured for 14 days post-purification/transduction. Mock mice=animals transplanted with non-transduced HSPCs purified as above (unless differently stated); Tie2-GFP transplanted mice=animals transplanted with HSPCs transduced with Tie2-GFP or Tie2-GFP_miRT (Escobar et al, 2014) lentiviral particles; Tie2-IFNα transplanted mice=animals transplanted with HSPCs transduced with Tie2-IFNα_miRT (Escobar et al, 2014) lentiviral particles.

Peripheral blood, intrahepatic leukocytes and splenocytes analyses

Seven to 10 weeks post-transplant, the whole anti-coagulated blood of Mock/Tie2-GFP and Tie2-IFNα mice was collected from the retro-orbital plexus of anesthetized animals (isoflurane, 5% for induction and 2% for maintenance in 2L/minute oxygen). Complete cell counts were measured in whole blood collected in 1/10th volume of EDTA (45mg/ml; Sigma-Aldrich) utilizing an automated cell counter (HeCoVet, Seac-Radim). Circulating WBCs were obtained after red blood cell lysis with TQ-Prep workstation (Beckman-Coulter). Intrahepatic leukocytes (IHLs) were isolated from the liver of Tie2-GFP mice intrasplenically injected with NaCl or 5x10^3 CT26 at indicated time points as described previously (Sitia et al, 2011). In selected experiments, spleens of Tie2-GFP or Tie2-IFNα mice were removed at time of killing, smashed and filter through 40um nylon filter. The resulting cell suspension was incubated with Ammonium-Chloride-Potassium lysing buffer to deplete red blood cells and washed with cold PBS. The phenotype of circulating WBCs, IHLs or splenocytes was determined by flow cytometry, utilizing the following antibodies: CD11b-PE/Cy7 (clone M1/70, BD Pharmingen); Ly-6G-APC/Cy7 (clone 1A8, Biolegend); Ly-6C-PB (clone HK1.4, Biolegend); NKp46-PE (clone 29A1.4, Biolegend); CD3-PE/Cy7 (clone 145-211C, BD Pharmingen); CD8-PE/Cy7 (clone 53-6.7, Biolegend); CD4-eFluor450 (clone RM4-5, eBioscience); B220-Alexa 647 (clone RA3-6B2, Biolegend); CD44-PE/Cy5 (clone IM7, Biolegend); CD62L-APC/Cy7 (clone MEL-14, Biolegend); CD25-PE (clone PC61, Biolegend); CD69-APC/Cy7 (clone H1.2F3, Biolegend); PD1-APC (clone J43, Bioscience); 7AAD (Pharmingen); CD45R-APC (clone RA3-6B2, Biolegend); CD11c-PE/Cy7 (clone N418, eBioscience). Tie2-GFP transplanted mice were also analyzed for the expression of GFP in order to estimate the

percentage of GFP^+ circulating monocytes, identified as: $Cd11b^+/Ly6C^+/Ly6G^-/GFP^+$ cells. To assess VCN in Tie2-GFP and Tie2-IFNα mice, genomic DNA was isolated from about $3x10^5$ WBCs/mouse using the Maxwell 16 automated extractor (Promega), quantified and analyzed by quantitative real-time PCR as previously described (Escobar et al, 2014).

Immunohistochemistry

At time of autopsy for each mouse, different organs were sampled, fixed in zinc-formalin, processed, embedded in paraffin, cut and stained with hematoxylin/eosin or further processed for immunohistochemical analyses as previously described (Guidotti et al, 1995; Sitia et al, 2011). Immunohistochemical staining was performed utilizing the following antibodies: anti-F4/80 (clone A3-1, AbD Serotec); anti-RFP (rabbit polyclonal, ab62341 AbCam); anti-CD34 (clone MEC14.7, Biolegend); anti-Ki67 (clone SP6, Neomarkers); anti-CD3 (clone SP7, AbCam); anti-CD45R/B220 (clone RA3-6B2, BD Pharmingen). All images were acquired using the Aperio Scanscope CS2 system (Leica Biosystems). To obtain the highest level of accuracy, the areas occupied by the RFP positive lesions in the liver were manually identified by three different operators blinded to any other information, summed and divided over the total liver area considered for analysis (between $12.4mm^2$ and $44mm^2$ per liver section), utilizing the ImageScope software (Leica Biosystems). All other quantifications were performed by automated image analysis software through dedicated macros of the ImageScope program, customized following manufacturer's instructions (Leica Biosystems). The images shown were identified as representative area of interest within the total area of the

specimen analyzed and exported as ImageScope snapshots. Image processing was performed post-analysis by Phostoshop CS4 (Adobe Systems Software).

Immunofluorescence and confocal microscopy

At time of autopsy different liver samples per mouse were collected, fixed over-night in 4% paraformaldehyde, equilibrated in a sucrose gradient (10% sucrose in PBS, 30 minutes at room temperature; 20% sucrose in PBS, 30 minutes at room temperature; 30% sucrose in PBS, over-night at 4°C), embedded in OCT for quick freezing at -80°C and cryosectioned (18-20µm thickness). Before immunofluorescence staining, sections were incubated with blocking buffer (PBS added with 5% fetal bovine serum and 0.1% Triton X-100 [Sigma-Aldrich]) for 30 minutes at room temperature. All subsequent staining and washing steps were performed with staining buffer (PBS added with 2% fetal bovine serum and 0.1% Triton X-100). Immunofluorescence staining was performed utilizing the following antibodies: anti-GFP (rabbit polyclonal, A11122 Invitrogen) + anti-rabbit Alexa 488 (Invitrogen); anti-MMR (goat polyclonal, AF2535 R&D Systems) + anti-goat Alexa 647 (Invitrogen); anti-F4/80-PE (clone A3-1, AbD Serotec); anti-CD11b-Alexa 647 (clone M1/70; Biolegend); Hoechst 33342 (Invitrogen). Confocal images were acquired using a Leica TCS SP2 or SP8 confocal systems (Leica Microsystems) that are available at the SRSI Advanced Light and Electron Microscopy BioImaging Center (ALEMBIC). 15-18µm z-stacks were projected in 2D and processed using Fiji image processing software (Schindelin et al, 2012) and Photoshop CS4 (Adobe Systems Software).

RNA extraction and quantitative RT-PCR gene expression analyses

Total RNA was isolated from liver homogenates by phenol-chloroform extraction as previously described (Guidotti et al, 1995). The extracted RNA was subsequently retro-transcribed to cDNA using Moloney Murine Leukemia Virus Reverse Transcriptase (M-MLV RT, Promega) as previously described (Sitia et al, 2011). Quantitative real-time PCR analysis was performed utilizing the 7900HT Fast Real-Time PCR System (Applied Biosystems). Raw data were analyzed using the SDS 2.3 software (Applied Biosystems). Gene expression was determined as the difference between the threshold cycle (Ct) of the gene of interest (*Goi*) and the Ct of the glyceraldehyde-3-phosphate dehydrogenase (*Gapdh*) of the same sample (∆Ct). The fold-change expression (i.e. fold increase) of each *Goi* was calculated over its basal expression in the control sample by the formula $2^{-\Delta\Delta Ct}$. The gene expression analysis of Tie2-GFP and Tie2-IFNα mice was performed utilizing the FAM-MGB labeled TaqMan Gene Expression Assays (Applied Biosystems): angiopoietin 2 (*Angpt2*, Mm00545822_m1), interferon regulatory factor 7 (*Irf7*, Mm00516793_g1). The murine *Gapdh* VIC-MGB labeled probe was utilized as endogenous control (Applied Biosystems). The analysis of GFP/*Gapdh* expression levels displayed in Fig 1D and of 2'-5'-oligoadenylate synthetase 1 *Oas1*/*Gapdh* and in Fig 2 was performed by Syber Green Gene Expression Assay (Applied Biosystems) utilizing the following primers: GFP-Fwd 5'-ACAAGCAGAAGAACGGCATC-3'; GFP-Rev 5'-CGGTCACGAACTCCAGCA-3'; GAPDH-Fwd 5' TTCACCACCATGGAGAAGGC-3'; GAPDH-Rev 5'-GGCATGGACTGTGGTCATGA-3'; OAS1a-Fw 5'- ATGGAGCACGGACTCAGGA-3'; OAS1a-Rev 5'- TCACACACGACATTGACGGC-3'.

Magnetic resonance imaging (MRI)

CB6 mice, IFNα/βR$^{-/-}$ mice and C57BL/6 mice were subjected to *in vivo* abdominal MRI in order to detect liver and peritoneal metastases and quantify their overall volume. All MRI studies were performed with a horizontal 7-Tesla MR scanner (Bruker, BioSpec 70/30 USR, Paravision 5.1, Germany), equipped with a gradient system characterized by amplitude of 450/675 milliTesla/meter (mT/m), slew rate of 3400/4500 Tesla/meter/second (T/m/s) and a rise time of 140 milliseconds (ms), coupled with a dedicated 4 channels volumetric mouse body coil. All mice underwent imaging under inhalational anesthesia (Isoflurane, 5% for induction and 2% for maintenance in 2L/minute oxygen); mice were kept prone on a dedicated temperature control apparatus to prevent hypothermia and breathing rate and body temperature were continuously monitored (SA Instruments, Inc., Stony Brook, NY, USA). All mice were prepared to MRI with intravenous injection of gadoxetic acid (Gd-EOB-DTPA; Primovist, Bayer Schering Pharma) at a dose of 0.05μmol/g of body weight. Axial fat-saturated T2-weighted images (TurboRARE-T2: TR=3394ms, TE=33ms, voxel-size=0.125x0.09x0.8mm, averages=3) were acquired immediately after Gd-EOB-DTPA injection, followed by axial fat-saturated T1-weighted sequences (RARE-T1: TR=581ms, TE=8.6ms, voxel-size=0.125x0.07x0.8mm, averages=4) acquired during the hepatobiliary phase of Gd-EOB-DTPA enhancement (10 minutes after administration), as previously described (Sitia et al, 2012). Image post-processing was performed using an advanced image segmentation open-source software (Mipav, 5.3.4 version, Biomedical Imaging Research Services Section, ISL, CIT, National Institute of Health, USA). Two radiologists with high experience in clinical and preclinical MR abdominal imaging

identified liver lesions in consensus, based on slight hyper-intensity on T2 images coupled with hypo-intensity on contrast-enhanced hepatobiliary phase T1 images, blinded to any other information. Lesions were selected as regions-of-interest (ROIs) on each slice forming volumes-of-interest (VOIs; lesion area x slice thickness) in the entire sequence. The total CRC metastatic mass was derived summing up individual VOIs values that were semi-automatically provided by the software. All MRI studies were performed at the Preclinical MRI and Ultrasound Facility of Experimental Imaging Center of SRSI.

LCMV infection and related procedures

The Armstrong strain of LCMV was utilized (Iannacone et al, 2008). Eighty-four or 54 days after receiving saline or CT26 CRC cells, Mock-transduced or wt mice (CTRL) or Tie2-IFNα mice were intraperitoneally infected with 200 pfu of LCMV. Whole blood was collected at the indicated time points from the retro-orbital plexus of anesthetized mice and white blood cell, hematocrit and platelet values were measured with an automated cell counter (HeCoVet, Seac-Radim). Single-cell suspensions were prepared from whole blood harvested at day 8 post-infection as previously described (Iannacone et al, 2008). Cells were stained with Pacific Blue-conjugated anti-CD8 (clone 53-6.7; BD Pharmingen) and allophycocyanin-conjugated anti-IFNγ (clone XMG1.2; BD Pharmingen). LCMV-specific $CD8^+$ T cells were identified by immunostaining with Pacific Blue-conjugated anti-CD8 (clone 53-6.7; BD Pharmingen) and by Phycoerythrin-conjugated recombinant soluble dimeric H-2d/Ig fusion protein (BD Pharmingen) complexed with the immune-dominant H-2d-restricted LCMV NP 118-126 peptide

(Isogawa et al, 2005). Samples were analyzed by flow cytometry with FACS CantoII (BD Pharmingen) and data were processed using FlowJo software (Tree Star Inc.). The presence of LCMV genomes in serum collected at the same time points abovementioned was quantified by real-time PCR using the following LCMV-specific primers 5'-CTCCTTTCCCAAGAGAAGACTAAG-3' and 5'-TCCATTTGGTCAGGCAATAAC-3' as previously described (Iannacone et al, 2008). All infections were performed in designated BSL-2 or BSL-3 workspaces, in accordance with institutional guidelines.

Appendix References

Binder D, Fehr J, Hengartner H, Zinkernagel RM (1997) Virus-induced transient bone marrow aplasia: major role of interferon-alpha/beta during acute infection with the noncytopathic lymphocytic choriomeningitis virus. J Exp Med 185: 517-530

Brattain MG, Strobel-Stevens J, Fine D, Webb M, Sarrif AM (1980) Establishment of mouse colonic carcinoma cell lines with different metastatic properties. Cancer Res 40: 2142-2146

De Palma M, Venneri MA, Roca C, Naldini L (2003) Targeting exogenous genes to tumor angiogenesis by transplantation of genetically modified hematopoietic stem cells. Nat Med 9: 789-795

Escobar G, Moi D, Ranghetti A, Ozkal-Baydin P, Squadrito ML, Kajaste-Rudnitski A, Bondanza A, Gentner B, De Palma M, Mazzieri R et al (2014) Genetic engineering of hematopoiesis for targeted IFN-alpha delivery inhibits breast cancer progression. Sci Transl Med 6: 217ra213

Follenzi A, Naldini L (2002) HIV-based vectors. Preparation and use. Methods Mol Med 69: 259-274

Guidotti LG, Matzke B, Schaller H, Chisari FV (1995) High-level hepatitis B virus replication in transgenic mice. J Virol 69: 6158-6169

Iannacone M, Sitia G, Isogawa M, Whitmire JK, Marchese P, Chisari FV, Ruggeri ZM, Guidotti LG (2008) Platelets prevent IFN-alpha/beta-induced lethal hemorrhage promoting CTL-dependent clearance of lymphocytic choriomeningitis virus. Proc Natl Acad Sci U S A 105: 629-634

Isogawa M, Furuichi Y, Chisari FV (2005) Oscillating CD8(+) T cell effector functions after antigen recognition in the liver. Immunity 23: 53-63

Kuo TH, Kubota T, Watanabe M, Furukawa T, Teramoto T, Ishibiki K, Kitajima M, Moossa AR, Penman S, Hoffman RM (1995) Liver colonization competence governs colon cancer metastasis. Proc Natl Acad Sci U S A 92: 12085-12089

Kuruppu D, Christophi C, Bertram JF, O'Brien PE (1996) Characterization of an animal model of hepatic metastasis. J Gastroenterol Hepatol 11: 26-32

Rosenberg SA, Spiess P, Lafreniere R (1986) A new approach to the adoptive immunotherapy of cancer with tumor-infiltrating lymphocytes. Science 233: 1318-1321

Schindelin J, Arganda-Carreras I, Frise E, Kaynig V, Longair M, Pietzsch T, Preibisch S, Rueden C, Saalfeld S, Schmid B et al (2012) Fiji: an open-source platform for biological-image analysis. Nat Methods 9**:** 676-682

Sitia G, Aiolfi R, Di Lucia P, Mainetti M, Fiocchi A, Mingozzi F, Esposito A, Ruggeri ZM, Chisari FV, Iannacone M et al (2012) Antiplatelet therapy prevents hepatocellular carcinoma and improves survival in a mouse model of chronic hepatitis B. Proc Natl Acad Sci U S A 109**:** E2165-2172

Sitia G, Iannacone M, Aiolfi R, Isogawa M, van Rooijen N, Scozzesi C, Bianchi ME, von Andrian UH, Chisari FV, Guidotti LG (2011) Kupffer cells hasten resolution of liver immunopathology in mouse models of viral hepatitis. PLoS Pathog 7**:** e1002061

Sobin LH, Compton CC (2010) TNM seventh edition: what's new, what's changed: communication from the International Union Against Cancer and the American Joint Committee on Cancer. Cancer 116**:** 5336-5339
