# Supplementary material for: IFNα gene/cell therapy curbs colorectal cancer colonization of the liver by acting on the hepatic microenvironment
Source: EMBO Mol Med. 2016 Jan 14;8(2):155–70. doi: 10.15252/emmm.201505395 (PMC4734840; doi:10.15252/emmm.201505395)
Supplement: Supplementary file 2 — Expanded View Figures PDF [file EMMM-8-155-s002.pdf]

Expanded View Figures

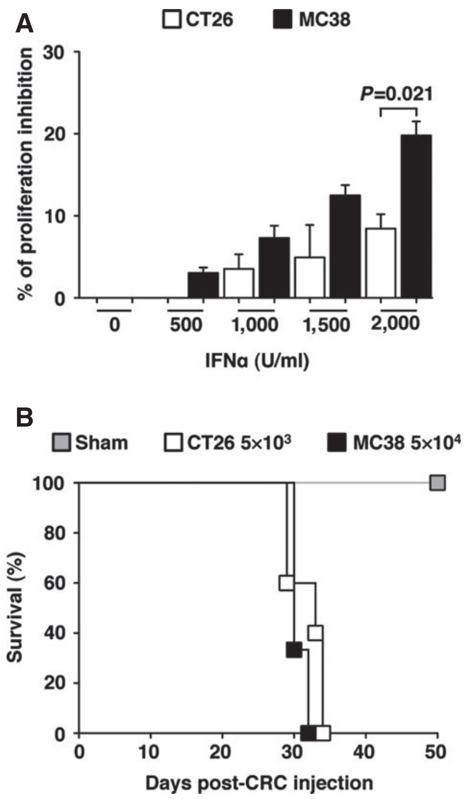

Figure EV1. Relative sensitivity of CRC cell lines to recombinant IFNα and survival of CB6 mice intrasplenically injected with different doses of CRC cells.

A The direct anti-proliferative effects of increasing doses of a recombinant murine IFNα on both CT26 and MC38 CRC cell lines were tested *in vitro* using a tetrazolium-based MTT assay. The percentage of proliferation inhibition was calculated after 72 h of stimulation by dividing the ODs at 570 nm of each IFNα-treated well by the relative ODs of the corresponding cell line left untreated. Mean values are shown; error bars indicate SEM; *P*-value was calculated by unpaired Student's *t*-test. Note that both cell lines are sensitive to the anti-proliferative action of recombinant IFNα with MC38 being slightly more sensitive at the highest concentration tested (CT26, n = 3 and MC38, n = 3; P = 0.03 and P = 0.01 for CT26 and MC38, respectively, by one-way ANOVA test, not reported on graph).

B Kaplan–Meier survival curves of wild-type CB6 mice intrasplenically injected with either 5×10^3 CT26 (n = 5) or 5×10^4 MC38 (n = 3) CRC cells. Note that this was the lowest dose of each CRC cell line that granted the highest metastatic incidence with comparable mortality rates in the two groups of animals. Sham, wt mice intrasplenically injected with NaCl (n = 2); data pooled from two independent experiments; differences between CT26- and-MC38 injected animals were not statistically significant by log-rank/Mantel–Cox test.

© 2016 The Authors

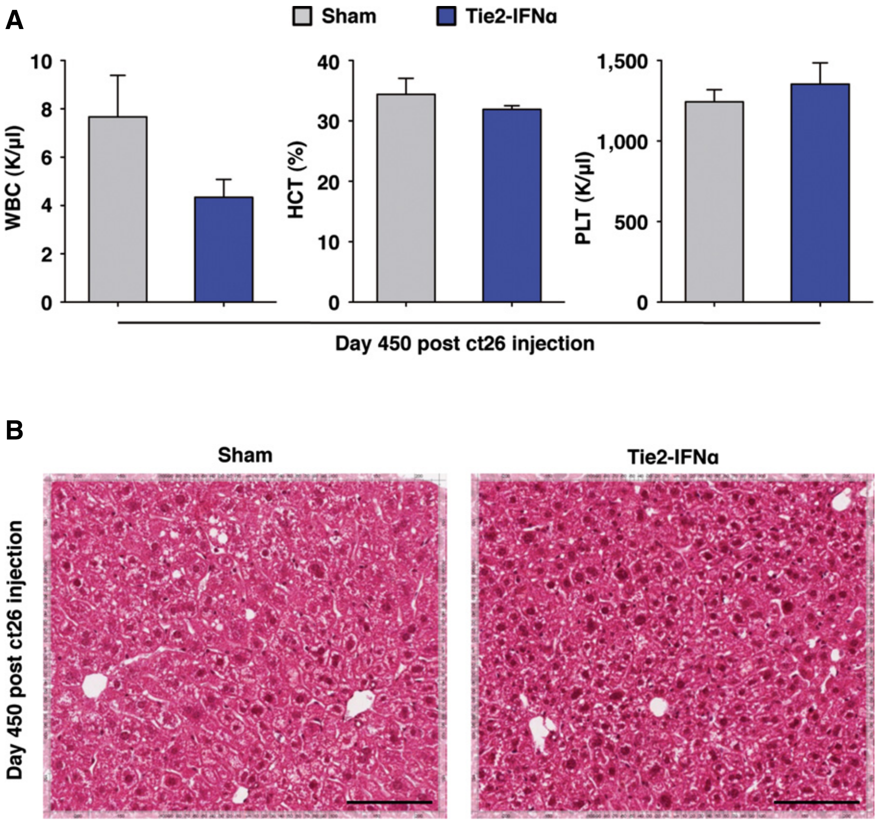

Figure EV2. Tie2-IFNα transplanted mice do not display signs of hematological toxicity or tumor relapse.

A White blood cell (WBC, left panel), hematocrit (HCT, middle panel), and platelet (PLT, right panel) counts of Sham (n = 3) and Tie2-IFNα (n = 5) mice described in Fig 2G (day 450 post-CRC injection). Despite the lower WBC count in Tie2-IFNα mice, which might have to be taken into account in longer-term studies, no statistically significant differences between the basic hematological values of the two groups were observed by unpaired Student's t-test; mean values are shown; error bars indicate SEM.

B Representative H&E micrographs from the liver of Sham (left panel) or Tie2-IFNα (right panel) mice described above. In both groups, the morphology of the hepatic tissue is consistent with the age of the mice. No signs of tumor relapse or hepatic damage are detectable in the liver of Tie2-IFNα mice at the time point analyzed (day 450); scale bars, 100 µm.

© 2016 The Authors

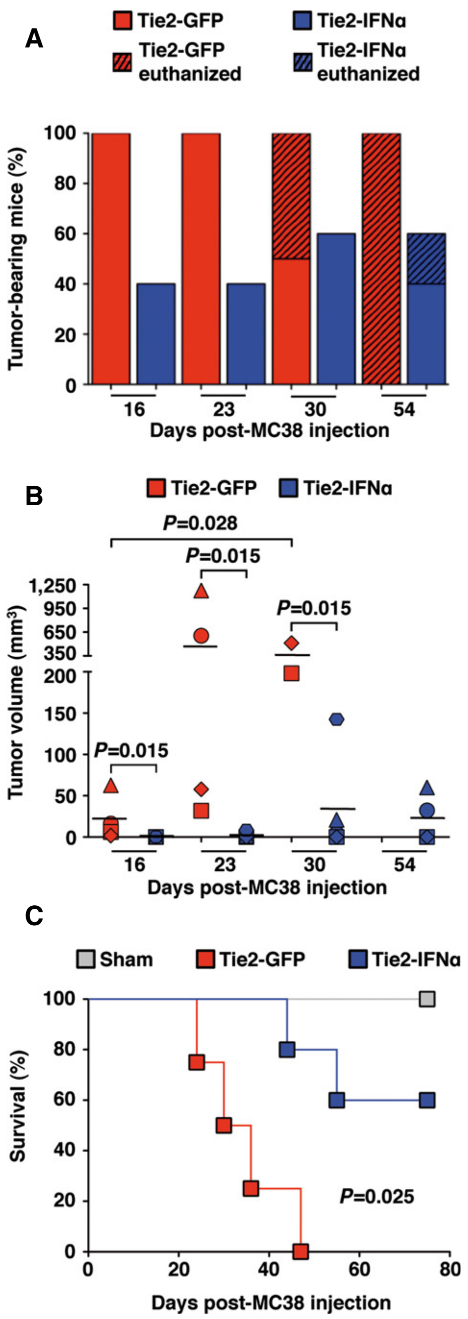

Figure EV3. Tie2-IFNα treatment impairs the metastatic growth of MC38 CRC cells in the liver.

A Percentage of CRC liver metastasis bearing mice estimated by MRI analysis. Tie2-GFP (n = 4) or Tie2-IFNα (n = 5) transplanted mice were intrasplenically injected with 5×10^4 MC38 CRC cells and analyzed at the indicated time points. The oblique black line pattern within the columns depicts the percentage of mice that died or were euthanized before the indicated time point; lnVCN (lineage-negative VCN calculated on cultured cells, see Appendix Supplementary Materials and Methods for details): Tie2-GFP = 7.6, Tie2-IFNα = 2.59.

B Tumor volume quantification of mice described in (A), estimated by MRI analysis as previously reported. Each symbol corresponds to an individual mouse analyzed at the indicated time points. Note that of the 3 Tie2-IFNα mice with liver metastases, the one depicted in Fig EV3B by a blue hexagon had to be euthanized at day 44 because of tumor growth. A second animal (depicted by a blue triangle) was subjected to liver MRI on day 54 and found dead on the following day; horizontal bars, mean values; P-values were calculated by Mann–Whitney U-test (to be able to perform the Mann–Whitney U-test at day 30, when some Tie2-GFP mice were euthanized/dead, we utilized tumor volume measured at day 23 from the same mice; no statistical analysis was performed at day 54).

C Kaplan–Meier survival curves of the indicated groups of mice described in (A). Note that the mouse, depicted by the blue triangle on the MRI of day 54, was found dead at day 55 presumably due to causes not related to tumor growth. Sham, Mock-transplanted mouse intrasplenically injected with NaCl (n = 1); P = 0.025 by log-rank/Mantel–Cox test.

© 2016 The Authors

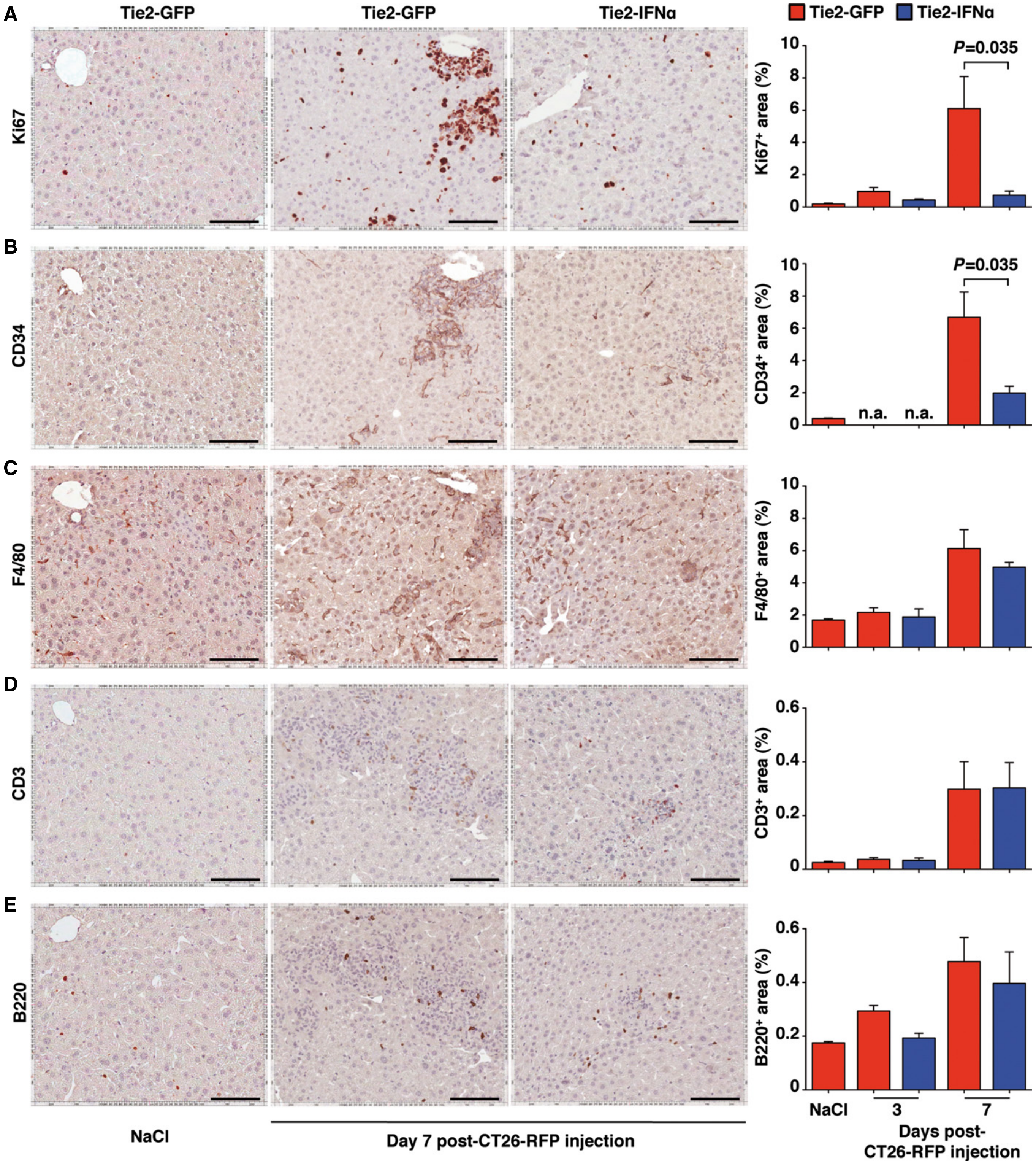

Figure EV4. TEM-mediated delivery of IFNα impairs tumor cell proliferation and signs of tumor-induced angiogenesis.

A–E Representative micrographs of the liver from Tie2-GFP mice (left and center panels) or Tie2-IFNα mice (right panels), 7 days post-intrasplenic injection of NaCl (left panels) or 5×10^5 CT26-RFP (center and right panels). The quantification of the percent hepatic area covered by each specific immunostaining at day 3 and 7 post-CT26-RFP injection is reported on the right (Tie2-GFP injected with NaCl $n = 2$, Tie2-GFP $n = 3$, Tie2-IFNα $n = 3$). Mean values are shown; error bars indicate SEM; n.a., not assessed; *P*-values were calculated by Mann–Whitney *U*-test. Scale bars, 100 μm. (A) Ki67, (B) CD34, (C) F4/80, (D) CD3, (E) B220 immunostaining.

 © 2016 The Authors

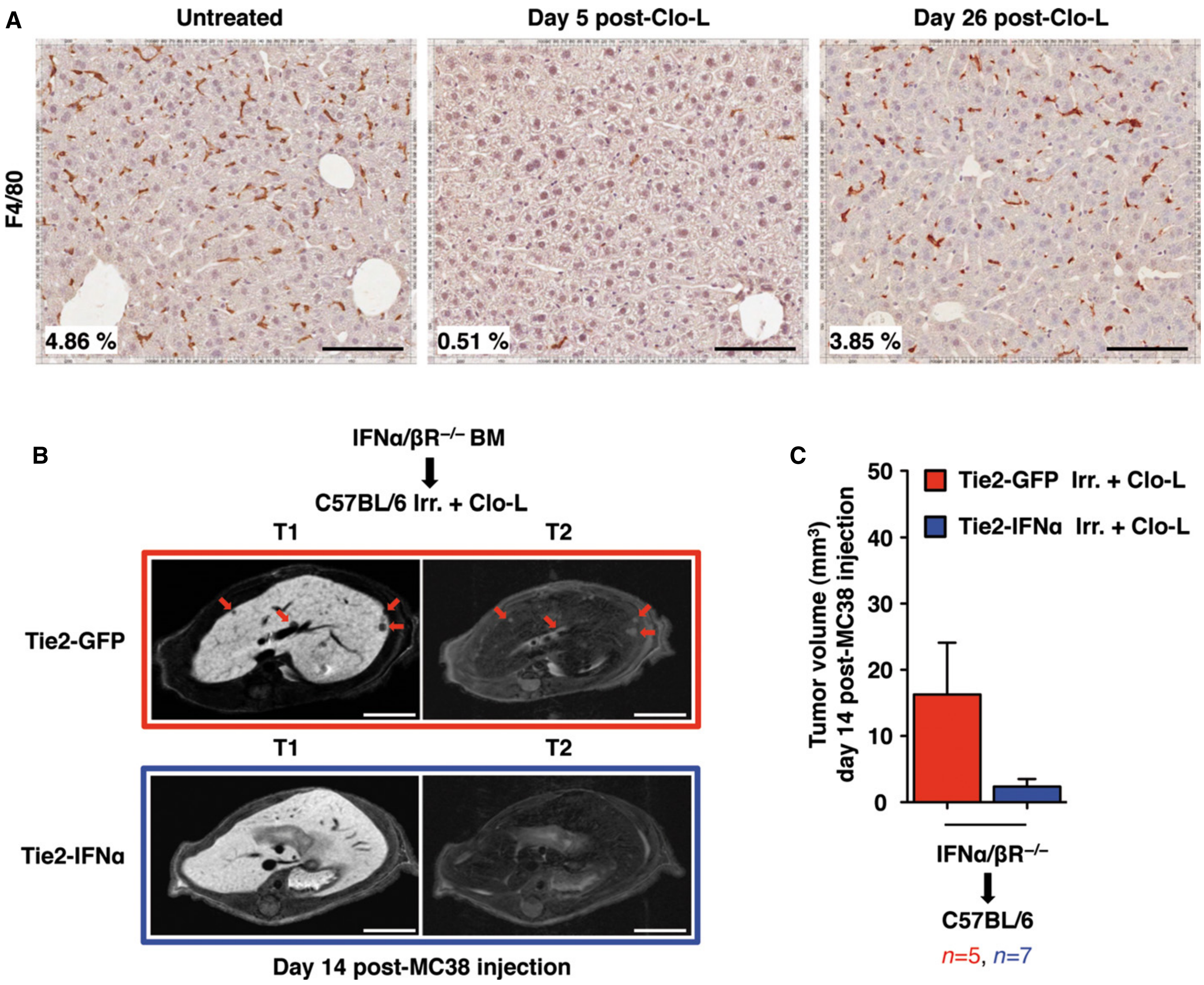

Figure EV5. Kupffer cells (KCs) are not primary targets of IFNα-mediated anti-tumor activity.

A Clodronate-containing liposomes (Clo-L)-mediated depletion of KCs in wild-type C57BL/6 mice and KCs liver repopulation (see Materials and Methods for details). Representative micrographs of the liver of C57BL/6 mice untreated (left panel) or injected intravenously with Clo-L (middle panel: day 5 post-administration; right panel: day 26 post-administration). For each condition, F4/80^{+} cells were identified by immunostaining and quantified as percentage of positive area (values are displayed in the bottom-left corner of each panel) of the liver section analyzed. Note that 26 days post-Clo-L administration the F4/80 signal is restored almost to normal levels; scale bars, 100 μm.

B Representative contrast-enhanced MRI panels of the liver of Tie2-GFP (red frame) or Tie2-IFNα (blue frame) chimeras, 14 days post-intrasplenic injection of 5×10^4 MC38. To generate chimeras having IFNα/βR$^{-/-}$ KCs, HSPCs were extracted from IFNα/βR$^{-/-}$ BM donor mice, transduced *in vitro*, and transplanted into lethally irradiated (Irr.) syngeneic C57BL/6 recipients. To deplete radio-resistant KCs (still sensitive to interferon) from recipient mice, 4 weeks post-transplant animals were treated with Clo-L as aforementioned. Subsequently, recipient mice were allowed to repopulate the liver with KCs derived from the transplanted HSPCs (lacking therefore the IFNα/β receptor) for additional 4 weeks. Finally, 8 weeks post-transplant mice were intrasplenically injected with 5×10^4 MC38 CRC cells as previously described. BM donor and recipient mouse strains are indicated on the top of the panels; red arrows identify CRC liver metastases of representative *z*-sections. Tumors are identified as hypointense and slightly hyperintense regions in T1- and T2-weighted sequences, respectively; scale bars, 5 mm.

C Tumor volume quantification estimated by MRI analysis of mice treated as described in (B). The chimeric group and the number of mice analyzed are listed; data pooled from two independent experiments; mean values are shown; error bars indicate SEM.

© 2016 The Authors
