# Supplementary material for: IFNα gene/cell therapy curbs colorectal cancer colonization of the liver by acting on the hepatic microenvironment
Source: EMBO Mol Med. 2016 Jan 14;8(2):155–70. doi: 10.15252/emmm.201505395 (PMC4734840; doi:10.15252/emmm.201505395)
Supplement: Supplementary file 3 — Movie EV1 [file EMMM-8-155-s003.zip › Movie_EV1/Movie_EV1_Legend.rtf]

Movie EV1. The movie shows T1-weighted MRI sequences performed at different time points encompassing the whole liver (in a cranial to caudal direction) of a representative Tie2-GFP mouse intrasplenically injected with 5x10^3^ CT26 described in the left panels of Fig 2A. Red arrows indicate hypointense regions identifying CRC liver metastases.
