# Supplementary material for: IFNα gene/cell therapy curbs colorectal cancer colonization of the liver by acting on the hepatic microenvironment
Source: EMBO Mol Med. 2016 Jan 14;8(2):155–70. doi: 10.15252/emmm.201505395 (PMC4734840; doi:10.15252/emmm.201505395)
Supplement: Supplementary file 4 — Movie EV2 [file EMMM-8-155-s004.zip › Movie_EV2/Movie_EV2_legend.rtf]

Movie EV2. The movie shows T1-weighted MRI sequences performed at different time points encompassing the whole liver (in a cranial to caudal direction) of a representative Tie2-IFNαmouse intrasplenically injected with 5x10^3^ CT26 described in the center panels of Fig 2A. No lesions are identifiable at the different time points analyzed. 
