# Supplementary material for: IFNα gene/cell therapy curbs colorectal cancer colonization of the liver by acting on the hepatic microenvironment
Source: EMBO Mol Med. 2016 Jan 14;8(2):155–70. doi: 10.15252/emmm.201505395 (PMC4734840; doi:10.15252/emmm.201505395)
Supplement: Supplementary file 7 — Movie EV5 [file EMMM-8-155-s007.zip › Movie_EV5/Movie_EV5_legend.rtf]

Movie EV5. Chimeric group: C57BL/6 HSPCs transplanted into IFNαβ-/- recipient. The movie shows T1-weighted MRI sequences, encompassing the whole liver (in a cranial to caudal direction) of 2 representative animals described in Fig 4A middle panels, performed 14 and 21 days post-intrasplenic injection of 5x10^4^ MC38 CRC cells. Red arrows indicate hypointense regions identifying CRC liver metastases.
