# Supplementary material for: IFNα gene/cell therapy curbs colorectal cancer colonization of the liver by acting on the hepatic microenvironment
Source: EMBO Mol Med. 2016 Jan 14;8(2):155–70. doi: 10.15252/emmm.201505395 (PMC4734840; doi:10.15252/emmm.201505395)
Supplement: Supplementary file 9 — Movie EV7 [file EMMM-8-155-s009.zip › Movie_EV7/Movie_EV7_Legend.rtf]

Movie EV7. Chimeric group: IFNαβ-/- HSPCs transplanted into C57BL/6 recipient. To deplete radio-resistant Kupffer cells still sensitive to IFNα recipient mice were treated with clodronate-containing liposomes (Clo-L) as previously described (see Fig EV5B and Materials and Methods for details). The movie shows T1-weighted MRI sequences, encompassing the whole liver (in a cranial to caudal direction) of 2 representative animals described in Fig EV5B, performed 14 days post-intrasplenic injection of 5x10^4^ MC38 CRC cells. Red arrows indicate hypointense regions identifying CRC liver metastases.
