# Supplementary material for: IFNα gene/cell therapy curbs colorectal cancer colonization of the liver by acting on the hepatic microenvironment
Source: EMBO Mol Med. 2016 Jan 14;8(2):155–70. doi: 10.15252/emmm.201505395 (PMC4734840; doi:10.15252/emmm.201505395)
Supplement: Supplementary file 10 — Movie EV8 [file EMMM-8-155-s010.zip › Movie_EV8/Movie_EV8_Legend.rtf]

Movie EV8. The movie shows T1-weighted MRI sequences performed at day 28 post-transplantation encompassing the whole liver (in a cranial to caudal direction) of representative Tie2-GFP and Tie2-IFNαmice intrahepatically injected with 5x10^3^ CT26 described in the Fig 5C. Red arrows indicate hypointense regions identifying CRC liver metastases, green arrows indicate peritoneal spreading of the disease (peritoneal carcinomatosis).
